# Greenhouse Gas Emissions from Three Cage Layer Housing Systems

**DOI:** 10.3390/ani2010001

**Published:** 2011-12-27

**Authors:** Sébastien Fournel, Frédéric Pelletier, Stéphane Godbout, Robert Lagacé, John Feddes

**Affiliations:** 1 Department of Soil Science and Agri-Food Engineering, Université Laval, 2425 Agriculture Street, Québec City, QC, G1V 0A6, Canada; E-Mail: robert.lagace@fsaa.ulaval.ca; 2 Research and Development Institute for the Agri-Environment, 2700 Einstein Street, Québec City, QC, G1P 3W8, Canada; E-Mails: sebastien.fournel@irda.qc.ca (S.F); frederic.pelletier@irda.qc.ca (F.P); 3 Department of Agricultural, Food and Nutritional Science, University of Alberta, Edmonton, AB, T6G 2P5, Canada; E-Mail: john.feddes@ualberta.ca

**Keywords:** laying hen, housing, greenhouse gas, emission, deep-pit, belt

## Abstract

**Simple Summary:**

Greenhouse gas (GHG) emissions were measured from three different cage layer housing systems. A comparative study was conducted to identify the housing system with the least impact on the environment. The results showed that liquid manure from deep-pit housing systems produces greater emissions of carbon dioxide (CO_2_), methane (CH_4_) and nitrous oxide (N_2_O) than natural and forced dried manure from belt housing systems. The influencing factors appeared to be the manure removal frequency and the dry matter content of the manure.

**Abstract:**

Agriculture accounts for 10 to 12% of the World’s total greenhouse gas (GHG) emissions. Manure management alone is responsible for 13% of GHG emissions from the agricultural sector. During the last decade, Québec’s egg production systems have shifted from deep-pit housing systems to manure belt housing systems. The objective of this study was to measure and compare carbon dioxide (CO_2_), methane (CH_4_) and nitrous oxide (N_2_O) emissions from three different cage layer housing systems: a deep liquid manure pit and a manure belt with natural or forced air drying. Deep liquid manure pit housing systems consist of “A” frame layer cages located over a closed pit containing the hens’ droppings to which water is added to facilitate removal by pumping. Manure belt techniques imply that manure drops on a belt beneath each row of battery cages where it is either dried naturally or by forced air until it is removed. The experiment was replicated with 360 hens reared into twelve independent bench-scale rooms during eight weeks (19–27 weeks of age). The natural and forced air manure belt systems reduced CO_2_ (28.2 and 28.7 kg yr^−1^ hen^−1^, respectively), CH_4_ (25.3 and 27.7 g yr^−1^ hen^−1^, respectively) and N_2_O (2.60 and 2.48 g yr^−1^ hen^−1^, respectively) emissions by about 21, 16 and 9% in comparison with the deep-pit technique (36.0 kg CO_2_ yr^−1^ hen^−1^, 31.6 g CH_4_ yr^−1^ hen^−1^ and 2.78 g N_2_O yr^−1^ hen^−1^). The shift to manure belt systems needs to be encouraged since this housing system significantly decreases the production of GHG.

## 1. Introduction

Worldwide environmental issues are dominated by climate change, especially by the increase in greenhouse gas (GHG) emissions [1]. The rise of GHG concentrations in the atmosphere has become a major environmental concern as revealed in the Kyoto Protocol [2]. Besides contributing to global warming by absorbing infrared radiation, carbon dioxide (CO_2_), methane (CH_4_) and nitrous oxide (N_2_O) have been declared as the most harmful gases for ecosystems, apart from ammonia (NH_3_) [3,4]. Agriculture accounts for 10 to 12% of the World’s total GHG emissions [5]. Manure management alone is responsible for 13% of GHG emissions from the agricultural sector [6].

Over the last decade, Québec’s egg production has shifted from deep-pit housing systems (liquid manure management) to manure belt housing systems (solid manure management). After reaching more than 90% in 1999, deep liquid manure pit systems have dropped to 36% in layer houses in 2009, while manure belt houses have become more popular increasing from 8 to 63% during the same period [7,8]. The same phenomenon has also been noted in the United States where newly constructed houses mostly use manure belt systems [9].

Deep liquid manure pit systems consist of “A” frame layer cages located over a closed deep pit containing the hens’ droppings to which water is added to facilitate removal by pumping. Generally, liquid manure is removed annually and stored in liquid manure tanks.

In the manure belt housing systems, fresh manure drops onto a conveyor belt beneath each row of battery cages. Manure on the belt is either dried by ambient air or by a forced-air stream through a perforated duct under the cages directed towards the stored manure. The moisture content of manure from belt systems varies from 60% with natural drying (ND) to 30% for forced air drying (FAD). Removal of manure ranges from daily to weekly intervals and occurs at one end of the house where manure can be stored (on- or off-farm) or directly applied to crop land.

Emissions of CO_2_, CH_4_ and N_2_O from these kinds of layer buildings have been reported in the literature (Table 1). Nevertheless, only few values are available for each housing system and they do not account for all the GHG. The differences observed for any range of values depend on the experimental context: climatic conditions, manure removal frequency, characteristics of hens, *etc.* The original values from the literature were converted to a kg or g yr^−1^ hen^−1^ basis for comparison purposes.

**Table 1:** Carbon dioxide (kg yr^−1^ hen^−1^), methane (g yr^−1^ hen^−1^) and nitrous oxide (g yr^−1^ hen^−1^) emissions from cage layer houses.

Greenhouse gas		Emissions ^a^		
		Unknown system with battery cages	Manure belt with forced air drying	Deep-pit system
CO_2_	n ^b^	2		
	Mean	26.5		
	Range	12.6–37.8		
	Reference	[10,11]		
CH_4_	n ^b^	3	2	3
	Mean	48.7	67.8	30.5
	Range	4.0–60.0	46.8–80.0	28.9–32.6
	Reference	[11,12,13]	[14,15]	[14,16]
N_2_O	n ^b^	3		1
	Mean	13.3		16.3
	Range	13.1–30.0		
	Reference	[10,17,18]		[16]

^a^ Values represent the converted emissions calculated by the authors. When the bird weight was unknown, it was estimated at 1.8 kg hen^−1^[19]. ^b^ n: number of values found in the literature.

In animal housing, the majority of CO_2_ emissions are generated from animal respiration (96%) and the remainder is from microbial activity in the animal’s digestive tract [6,20,21]. CH_4_ originates from the anaerobic decomposition of organic compounds during enteric fermentation, a digestive process that occurs mainly in ruminants or during manure storage [6,14,20,22]. For laying hens, CH_4_ emissions from enteric fermentation are expected to be negligible thus emissions are primarily from manure stored under anaerobic conditions [14]. N_2_O is an intermediate product from nitrification and denitrification processes under conditions of low oxygen availability [14,17,20,23,24].

The general objective of this study was to measure and compare GHG emissions from three different cage layer houses: a deep liquid manure pit system and a manure belt system with ND or FAD. The different systems will be compared under controlled conditions to identify the system with the lowest environmental impact.

## 2. Material and Methods

### 2.1. Experimental Rooms

**Figure 1:**
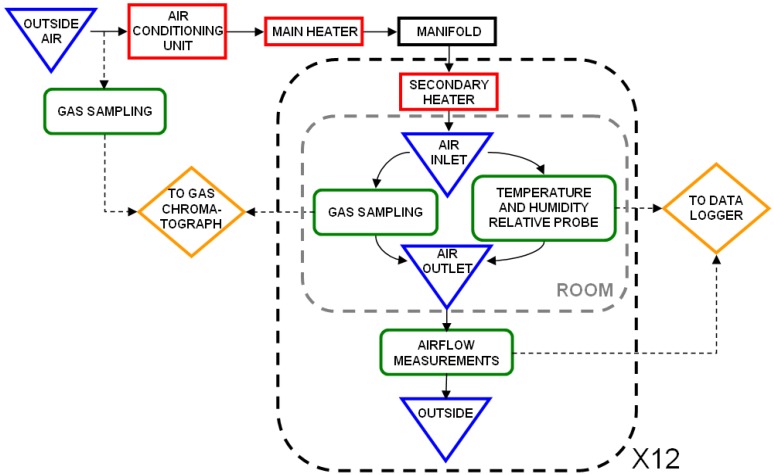
Schematic view of the air sampling in the laboratory.

The experiment was conducted in a laboratory (Figure 1) which consists of twelve independent bench-scale rooms (1.2 m wide × 2.4 m long × 2.4 m high), arranged side by side. Each room is equipped with a variable speed exhaust fan. The incoming air, drawn from outside the laboratory, is the same for all the rooms and comes from a main duct where it is pre-conditioned. An air conditioning unit is used, if necessary, to cool the air before entering the rooms. During the cold season, the air is heated by a heating system located just after the air conditioner. A second heating unit, located in the ventilation duct of each room, allows an optimal temperature adjustment for each chamber.

### 2.2. Housing Systems

**Figure 2:**
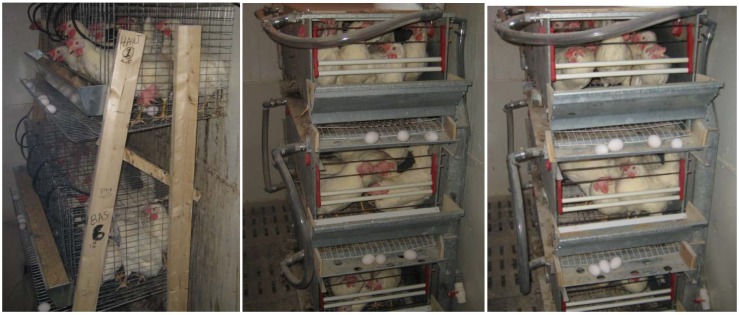
Deep liquid manure pit system (left) and manure belt systems with natural (center) and forced air drying (right).

Three hundred and sixty hens (30 per room) were housed in three different housing systems (Figure 2):

(1) Deep liquid manure pit system—Hens were confined to a commercial cage system (Ranch Cunicole G.L.R. Inc., Saint-Hyacinthe, QC, Canada) measuring 1,524 mm long, 457 mm deep and 457 mm high. The two levels, assembled as an “A” frame, included five sections of 305 mm in length with three hens in each one (465 cm^2^ hen^−1^). Manure dropped beneath the cages into a pit at the bottom of the room where water was added to facilitate pumping the manure in a liquid form at the end of the trials;
(2) Manure belt system with ND—Hens were reared in Farmer Automatic’s Multi-Deck battery cages (485.5 mm wide × 507 mm deep × 540 mm high) placed 2 × 2 on three decks for a total of six cages. Each cage included five hens (492 cm^2^ hen^−1^). Manure dropped on a belt beneath each row of cages and was removed twice a week;
(3) Manure belt system with FAD—Manure dropped on a belt beneath each row of cages where it was dried with forced air and removed twice a week. The drying system was installed under all the decks of the Farmer Automatic’s battery cages. A perforated 7.5-cm duct blew air from a 10-cm blower (VTX-400, Atmosphere, Terrebonne, QC, Canada) located beneath the air inlet. Five-mm holes were placed at each 160 mm with a 45-degrees angle. Then, based on an air flow of 1.3 m^3^ h^−1^, the blower was adjusted to obtain an air velocity of 3.05 m s^−1^.

### 2.3. Animals, Lighting and Feeding

Laying hens were Lohmann LSL-Lite. The lighting period was 13 h d^−1^ for weeks 1 and 2, 13.5 h d^−1^ for weeks 3 and 4 and 14 h d^−1^ for the last four weeks. The lighting system was regulated to give 40 lux per room (Light Meter, Lux/FC, 840020C, Sper Scientific, Ltd., Scottsdale, AZ, USA). Water was provided by a solenoid activated valve connected to a data logger to register the water flow through the nipple drinkers inside the cages. The hens were fed 100 g hen^−1^ d^−1^ of a commercial diet (2005-000, Aliments Breton, St-Bernard-de-Beauce, QC, Canada).

### 2.4. Temperature and Relative Humidity

The temperature and relative humidity of the air in each individual room were measured using a probe (model CS500, accuracy ±0.5 °C at 20 °C and ±3% RH, Campbell Scientific Canada, Corp., Edmonton, AB, Canada ). A datalogger was connected to a computer to upload data coming from the temperature-relative humidity probe every 10 s and the average value was recorded every 15 min. In accordance with commercial practices, the temperature in the rooms was set at 22.5 °C.

### 2.5. Ventilation Rates

Ventilation rates were calculated from a 204-mm iris orifice damper (Model 200, accuracy ±5%, Continental Fan Manufacturing Inc., Buffalo, NY, USA) installed in the exhaust duct of each room. The difference of pressure was measured across the damper every 10 s and a data logger recorded the average every 15 min during each trial. The average value was then used to calculate the ventilation rate using Equation (1) as follows:

$$Q=k\sqrt{∆pressure}\times 0.0283$$ (1)

where *Q* is the ventilation flow rate for one room during one sampling event (m^3^ min^−1^), *k* is a constant which depends on the setting of the iris damper, *Δpressure* is the difference in static pressure (inches of water) and 0.0283 is a conversion factor to obtain the desired SI units.

### 2.6. Composition of Manure

Manure samples were collected every week of each trial. A fixed amount of manure was taken from a random spot in each room. In the deep-pit rooms, the samples were taken from the pit under the floor. In the manure belt houses, the samples were taken on the belt. The samples were analysed for dry matter content (DMC), pH, total nitrogen (TN), ammonium nitrogen (NH_4_-N) and minerals (P, K, Ca and Mg).

### 2.7. Greenhouse Gas Concentrations and Emissions

The sampling air was pumped to a mobile laboratory through Teflon™ tubing. In this laboratory, CO2, CH4 and N2O were analyzed by gas chromatography (GC) (model 3600, Varian, Walnut Creek, CA, USA). The strategy for chromatographic analysis was the separation of the three gases in columns packed with Porapak Q (Waters Corporation, Milford, MA, USA). The CH4 was quantified with a flame ionisation detector while the CO2 and N2O were measured with an electron capture detector. The instrumental errors on CO2, CH4 and N2O concentrations were ±30, 0.5 and 0.1 ppmv.

Samples were pumped from the experimental rooms through the injection loop of the GC for 15 min, after which the sample was analysed by the GC. A multiport valve was used to cycle gas sampling between the different points every 15 min. Concentration measurements were taken continuously during the entire experiment and were synchronized with the ventilation flow rate. Every three days between 12:00 PM and 1:00 PM, a standard gas containing known concentrations of the three GHG was analyzed to verify the response of the GC. Dust filters were installed before each trial at the inlet of each Teflon™ sampling line to avoid contamination and damage to the gas analyzers.

The GHG emissions were calculated for each sampling period using Equation (2) as follows:

$$E_{GHG}=(C_{out}-C_{in})\times \frac{Q}{N_{hens}}\times \frac{P_{atm}-P_{v}}{287\times T}\times \frac{M_{GHG}}{M_{air}}\times 525.6$$ (2)

where *E_GHG_* represents CO_2_, CH_4_ or N_2_O emissions for one room during one sampling event (g yr^−1^ hen^−1^), *C_in_* is the GHG concentration at the room inlet (ppmv), *C_out_* is the GHG concentration at the exhaust fan of the room (ppmv), *N_hens_* is the number of hens in the room, *P_atm_* and *P_v_* are respectively the atmospheric pressure at sea level and the vapor pressure in the room (Pa), *T* corresponds to the temperature (K), *M_GHG_* characterizes the molar masses of CO_2_ (44.0 g mol^−1^), CH_4_ (16.0 g mol^−1^) or N_2_O (44.0 g mol^−1^), *M_air_* signifies the molar mass of air (28.97 g mol^−1^), 287 is the thermodynamic constant of air (J kg^−1^ K^−1^) and 525.6 is a conversion factor (mg min^−1^ to g yr^−1^).

### 2.8. Experimental Design and Statistical Analysis

The experiment was carried out over two successive 8-wk trials (March–May 2010 and June–August 2010) corresponding to the beginning of the egg laying period (19 to 27 weeks of age). This 8-wk period was found to produce the highest rate of gas emissions [25]. An 11-day acclimatization period preceded each trial. The four repetitions of each housing system were randomly assigned to the experimental rooms.

Consequently, the experiment was a completely randomized design with three housing systems and four repetitions, repeated twice. Weekly averages were calculated from all emissions measured for each period of 15 min within one week and reported in kg or g yr^−1^ hen^−1^ to facilitate comparison with the literature. The measurements made during the eight weeks of a same room are considered as being correlated. The analysis of variance with repeated measurements in time requires the use of a mixed model to analyze the effect of the systems on CO_2_, CH_4_ and N_2_O emissions. The invariable effects of the model are the housing system, the week and the system interaction by week. Random effects are the trial, the trial interaction by system by repetition due to the variation between the rooms for each trial and the residual error. The procedure PROC MIXED of the SAS program (Version 8, 1999, SAS Institute, Inc., Cary, NC, USA) was used to adjust the model [26].

## 3. Results and Discussion

### 3.1. Environmental Parameters

The outside temperature (12.4 °C for trial 1 *vs.* 22.3 °C for trial 2) between both trials was different due to the change of season (March–May for trial 1 *vs.* June–August for trial 2). This seasonal effect influenced the average ventilation flow rates as well since they were slightly greater during trial 2 (1.03 L s^−1^ hen^−1^ for trial 1 *vs.* 1.19 L s^−1^ hen^−1^ for trial 2). The minimum and maximum ventilation flow rates ranged from 0.13 to 1.98 L s^−1^ hen^−1^ for trial 1 and from 0.54 to 3.00 L s^−1^ hen^−1^ for trial 2. This corresponds to the recommended flow rates in Québec with 0.2 L s^−1^ hen^−1^ in winter and 3.3 L s^−1^ hen^−1^ in summer. Despite small flow differences between rooms within each trial, the average temperature in the rooms was considered constant indicating a proper operation of the environmental control equipment. The change of season only resulted in a small temperature difference between both trials (22.1 °C for trial 1 *vs.* 22.4 °C for trial 2).

### 3.2. Performance of the Laying Hens

The hens reared in deep-pit houses consumed more (*P* < 0.05) feed (105.5 g d^−1^) and water (0.174 L d^−1^) as compared to those housed in the rooms with manure belt systems (about 93 g d^−1^ and 0.162 L d^−1^) (Table 2). The different configuration of the cages (lighting angle, number of animals per cage, *etc.*) could explain this difference. In spite of the higher consumption, the hens in deep-pit rooms didn’t weigh more (*P* > 0.05) since the initial and final weights were similar between the three housing systems (1.37 and 1.64 kg, respectively). Because of the higher feed consumption, these hens had the lowest conversion rate (1.34 kg of food consumed per dozen of eggs produced). The efficiency of egg production was improved by 12% by using the manure belt housing systems instead of deep liquid manure pit systems.

### 3.3. Manure Characteristics

The mass of manure produced by the deep-pit hens (140 g d^−1^ hen^−1^) was higher (*P* < 0.05) than the ND manure (71 g d^−1^ hen^−1^) and the FAD manure (61 g d^−1^ hen^−1^) due to the addition of water at the beginning and at the end of each trial (Table 3). This water addition also resulted in a lower DMC (*P* < 0.05). However, the liquid manure contained more dry matter when the amount of manure is reported on a dry-matter basis. The higher feed consumption from the hens in the deep-pit system could explain this result (Table 2). Besides, the difference in mass (*P* > 0.05) and DMC (*P* < 0.05) between the two other systems is due to the forced drying system which increases evaporation from the manure.

**Table 2:** Performance of the laying hens in the three housing systems.

Parameter		Deep liquid manure pit		Manure belt—natural drying		Manure belt—forced air drying	
		Mean ^a^	SD	Mean ^a^	SD	Mean ^a^	SD
Initial weight	kg hen^−1^	1.38 a	0.04	1.37 a	0.04	1.36 a	0.06
Final weight	kg hen^−1^	1.64 a	0.06	1.65 a	0.03	1.63 a	0.03
Food consumption	g d^−1^ hen^−1^	105.5 a	3.4	93.1 b	1.5	92.7 b	2.0
Water consumption	L d^−1^ hen^−1^	0.174 a	0.018	0.160 b	0.008	0.163 b	0.014
Egg production	egg d^−1^ hen^−1^	0.943 b	0.015	0.950 a	0.020	0.932 c	0.027
Conversion rate	kg_food_ dz_eggs_^−1^	1.34 a	0.06	1.18 a	0.03	1.19 a	0.04

^a^ Mean values followed by the same letter in a row are not significantly different at *P *= 0.05 as determined by pairwise contrasts. SD: standard deviation.

During a study on 24 facilities in Québec, Seydoux *et al.* [27] noted a 12-percent DMC for liquid manures and a 55-percent DMC for ND and FAD manures. However, the reported values differ from these values. In the case of liquid manures, Seydoux *et al.* [27]’s samples were taken directly from the liquid manure tanks where dilution by rain could occur. This may have resulted in a lower DMC of manure samples. Nevertheless, it should be noted that a DMC of 10%, near what Seydoux *et al.* [27] proposed, was obtained at the beginning of each trial with the addition of water. However, it increased quickly to about 30% during the subsequent weeks possibly because of suspected leaks in the storage pit. The inability to retain all the liquid thus resulted in an average DMC of 23%, which is nonetheless comparable to what Lockyer *et al.* [28] found for droppings stored beneath the cages in a pit where water was added (26-percent DMC). In the case of ND and FAD manures, even though Fabbri *et al.* [14] obtained a DMC similar to 44% for the ventilation belt technique (28 to 42%), they considered this result lower than that generally reached with this method. Actually, the TS content of dried manure should be around 50 to 70% [9,27,28,29,30]. Higher values of DMC could have been obtained with the drying system in this study, but several short (less than 24 h) power failures occurred, probably resulting in lower DMC. Besides, some producers in Québec increase the heating during winter for greater manure drying. This operation could have influenced the results from Seydoux *et al.* [27].

For the deep-pit system, TN was lower while NH_4_-N was clearly higher than in the other two housing systems. With manure drying, higher losses of nitrogen through NH_3_ emissions should have resulted in lower concentrations in manure [25]. On the other hand, the liquid manure contained more NH_4_-N because of the higher moisture content which favoured microbial conversion of urea to NH_4_-N. Liquid manure analyses also resulted in lower phosphorus (*P* < 0.05), potassium (*P* < 0.05), magnesium (*P* > 0.05) and calcium (*P* > 0.05) contents. Similar mineral contents were found by Seydoux’s *et al.* [27] for nitrogen (1.11 to 1.5 g d^−1^ hen^−1^), phosphorus (0.42 to 0.48 g d^−1^ hen^−1^), potassium (0.52 to 0.64 g d^−1^ hen^−1^), calcium (1.4 to 1.63 g d^−1^ hen^−1^) and magnesium (0.13 to 0.134 g d^−1^ hen^−1^).

**Table 3:** Composition of the manure collected in the deep pit (liquid manure) and on the manure belts (natural or forced dried manures).

Performance parameter			Liquid manure		Natural dried manure		Forced dried manure	
			Mean ^a^	SD	Mean ^a^	SD	Mean ^a^	SD
Manure quantities		g d^−1^ hen^−1^	140.2 a	6.5	70.9 b	7.5	60.8 b	7.3
		g DM d^−1^ hen^−1^	32.3 a	1.5	26.2 a	2.8	26.6 a	3.2
Dry matter content	DMC	%	23.0 c	2.0	37.0 b	2.8	43.8 a	4.4
pH			7.59 a	0.12	6.66 b	0.14	6.76 b	0.08
Total nitrogen	TN	g d^−1^ hen^−1^	0.97 b	0.13	1.70 a	0.12	1.64 a	0.13
Ammonium nitrogen	NH_4_-N	g d^−1^ hen^−1^	0.52 a	0.10	0.26 ab	0.05	0.19 b	0.02
Phosphorus	P	g d^−1^ hen^−1^	0.33 b	0.03	0.46 a	0.04	0.44 a	0.04
Potassium	K	g d^−1^ hen^−1^	0.45 b	0.02	0.61 a	0.04	0.59 ab	0.05
Calcium	Ca	g d^−1^ hen^−1^	1.39 a	0.08	1.83 a	0.06	1.81 a	0.14
Magnesium	Mg	g d^−1^ hen^−1^	0.105 a	0.004	0.144 a	0.006	0.138 a	0.011

^a^ Mean values followed by the same letter in a row are not significantly different at *P *= 0.05 as determined by pairwise contrasts. SD: standard deviation.

### 3.4. Carbon Dioxide Emissions

The deep liquid manure pit and the manure belt systems with ND and FAD emitted respectively 36.0, 28.2 and 28.7 kg CO_2_ yr^−1^ hen^−1^ (Table 4). Therefore, the ND and FAD manure belt systems reduced CO_2_ emissions between 20 and 22% although 96% of CO_2_ is produced by animal respiration [21]. Consequently, the significant (*P* < 0.05) difference in CO_2_ emissions is due to the remaining 4% attributable to manure decomposition. Long-term storage of manure inside the deep-pit rooms allows a more intensive degradation of manure into CO_2_. No significant difference (*P * > 0.05) was found between the manure belt systems.

**Table 4:** Mean carbon dioxide (kg yr^−1^ hen^−1^), methane (g yr^−1^ hen^−1^) and nitrous oxide (g yr^−1^ hen^−1^) emissions from the three housing systems.

Greenhouse gas	Emissions ^a^					
	Deep liquid manure pit		Manure belt— natural drying		Manure belt— forced air drying	
	Mean	SD	Mean	SD	Mean	SD
CO_2_	36.0 a	8.3	28.2 b	7.1	28.7 b	6.3
CH_4_	31.6 a	22.9	25.3 b	19.2	27.7 b	20.2
N_2_O	2.78 a	2.15	2.60 a	2.04	2.48 a	1.91

^a^ Means values followed by the same letter in a row are not significantly different at *P *= 0.05 as determined by pairwise contrasts. SD: standard deviation.

**Figure 3:**
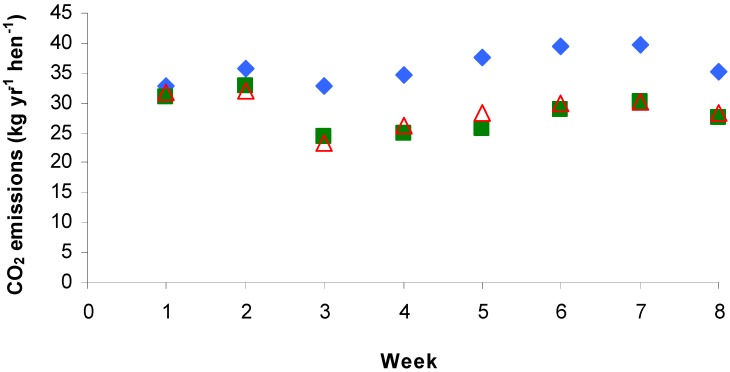
Mean weekly carbon dioxide emissions from three housing systems: deep liquid manure pit (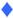), manure belt with natural drying (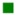) and manure belt with forced air drying (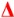).

Furthermore, the statistical analysis showed a significant week effect on CO_2_ emissions (*P* < 0.05), as shown in Figure 3. In fact, since the CO_2_ production is directly proportional to laying hens weight, the production should have increased over time as they got heavier, as seen in Wu-Haan *et al.* [11]. Although the emission factors increased between weeks 3 to 7, the CO_2_ production was higher during weeks 1 and 2. A possible explanation could be the change in the partitioning of energy as the birds progress from first egg to peak lay [31,32,33,34].

The mean housing CO_2 _emissions were similar to the results obtained by Neser *et al.* [10] and Wu-Haan *et al.* [11] with laying hens reared in battery cages (12.61 to 37.84 and 23.95 to 32.57 kg yr^−1^ hen^−1^, respectively; Table 1).

### 3.5. Methane Emissions

The CH_4_ emissions from the deep liquid manure pit, the ND and FAD manure belt systems were 31.6, 25.3 and 27.7 g yr^−1^ hen^−1^ (Table 4). Therefore, the manure belt systems reduced CH_4_ emissions by 20 and 12%, respectively, compared to that of the deep-pit system. Since CH_4_ is a product of the anaerobic digestion of manure [14,20,22], the significant difference (*P* < 0.05) between the deep-pit technique and the manure belt systems is due to the favourable anaerobic conditions in liquid manure. The addition of the initial water to the manure resulted in a lower DMC value and a reduction of oxygen. Liang *et al.* [35] found that an accumulation of manure generates anaerobic conditions after 25 days. In addition, there was no difference (*P * > 0.05) between the ND and FAD manure belt systems.

Apart from the anaerobic conditions, pH could be also a parameter responsible for a higher CH_4_ production by the deep-pit system. It appears that CH_4_ emissions are greatest at a pH of 7 and that acidification could be a mitigation strategy to reduce emissions [36]. In fact, the CH_4_ emissions are reduced by 50% at pH 6.5. Accordingly, lower pH values (6.66 and 6.76, respectively; Table 3) for ND and FAD manures could have resulted in smaller CH_4_ emission rates relative to liquid manure (7.59; Table 3). In the study of Wu-Haan *et al.* [11], a change in manure pH from hens fed two different diets resulted in a 17% reduction in CH_4 _emissions. The slight difference in pH between the ND and FAD systems could also explain that the ND system resulted in smaller CH_4_ emissions even though FAD manure had a greater DMC, which is supposed to lower emissions [14].

**Figure 4:**
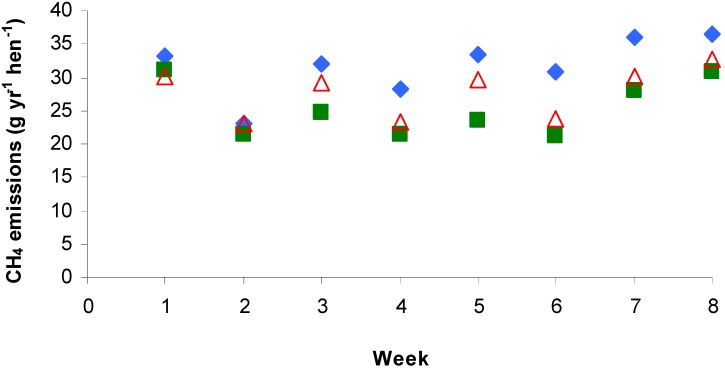
Mean weekly methane emissions from three housing systems: deep liquid manure pit (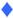), manure belt with natural drying (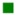) and manure belt with forced air drying (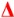).

A significant difference in CH­_4_ emissions was also found among the weeks for all the systems (*P* < 0.05). The same effect was also noted by Wu-Haan *et al.* [11] who found that 21-wk-old hens likely produce greater CH_4_ emissions than at 38 or 59 weeks of age. The results in this study could not confirm this tendency since emissions during weeks 7 and 8 were greater than those of weeks 2 to 6 (Figure 4). In addition, the difference between the first two weeks could be, as for CO_2_ emissions, the result of a change in the partitioning of energy for egg production or weight gain [31,32,33,34].

The measured values are in accordance with those of Fabbri *et al.* [14], Wathes *et al.* [16] and Wu-Haan *et al.* [11] who obtained CH_4_ emission rates between 28.94 and 32.56 g yr^−1^ hen^−1^ for similar deep-pit and FAD manure belt housing systems, respectively (Table 1). However, CH_4_ emissions twice that of those in this study (56 g yr^−1^ hen^−1^; Table 1) were observed by Groot Koerkamp and Uenk [12], Hörnig *et al.* [15] and Monteny *et al.* [13]. In the case of Hörnig *et al.* [15], their experiment took place in farm-scale barns, which resulted in large variations within the area. High ventilation rates could have also caused greater emissions, especially in summer where emission rates sometimes reached 320 g CH_4_ yr^−1^ hen^−1^.

### 3.6. Nitrous Oxide Emissions

The N_2_O emissions measured for the deep liquid manure pit, the ND and FAD manure belt housing systems were 2.78, 2.60 and 2.48 g yr^−1^ hen^−1^, respectively (Table 4). These results were statistically similar (*P * > 0.05). The measured N_2_O emissions are below those obtained by Chadwick *et al.* [17], Sneath *et al.* [18] and Wathes *et al.* [16] (13 to 30 g yr^−1^ hen^−1^; Table 1), but comparable to Neser *et al.* [10] (0.63 to 4.73 g yr^−1^ hen^−1^; Table 1). The results obtained here come from the very low N_2_O concentrations measured, only slightly higher than the concentrations in ambient air. The same observations established by some authors [14,16,37] indicated that N_2_O emissions from layer houses should be judged critically since they vary considerably due to the low concentrations from which emissions are calculated. In certain cases, no significant emissions were registered for N_2_O since they were consistently near the detection limit for deep-pit systems and manure belt systems [14]. Nevertheless, the very lowest N_2_O emissions should be taken into account due to their potential of global warming (310 times higher than CO_2_).

### 3.7. Greenhouse Gas Emissions

Total GHG emissions can be calculated on a CO_2_-equivalent basis (Table 5). The evaluation of GHG emissions is based only on manure emissions. In this case, since the majority of CO_2_ is produced by animal metabolism, only CH_4_ and N_2_O were considered. The FAD (1.35 kg CO_2_-eq. yr^−1^ hen^−1^) and ND (1.34 kg CO_2_-eq. yr^−1^ hen^−1^) manure belt housing systems reduced GHG emissions by 11.5 and 12.5% compared to the deep liquid manure pit houses (1.53 g CO_2_-eq. yr^−1^ hen^−1^). These results only consider the emissions at the barn level and could vary if other factors such as energy consumption were also taken into consideration.

**Table 5:** Greenhouse gas emissions from the three housing systems.

Gas		Emissions (kg CO_2_-eq. yr^−1^ hen^−1^)		
Name	Global warming potential	Deep liquid manure pit	Manure belt—natural drying	Manure belt—forced air drying
CH_4_	21	0.664	0.532	0.582
N_2_O	310	0.862	0.806	0.769
Total		1.53	1.34	1.35

## 4. Conclusions

The specific objective addressed in this research consisted in determining which of the three cage layer housing systems was the most promising in mitigating GHG emissions. The results demonstrated that FAD and ND manure belt systems reduced CO_2_ (28.2 and 28.7 kg yr^−1^ hen^−1^, respectively), CH_4_ (25.3 and 27.7 g yr^−1^ hen^−1^, respectively) and N_2_O (2.60 and 2.48 g yr^−1^ hen^−1^, respectively) emissions by about 21, 16 and 9% compared to the deep-pit technique (36.0 kg CO_2_ yr^−1^ hen^−1^, 31.6 g CH_4_ yr^−1^ hen^−1^ and 2.78 g N_2_O yr^−1^ hen^−1^). These values represent about 12% less GHG emissions in the atmosphere. Therefore, manure belt systems need to be encouraged since these techniques significantly decrease the production of GHG and have fewer effects on environment.
